# Trends in the Use of Naltrexone for Addiction Treatment among Alcohol Use Disorder Admissions in U.S. Substance Use Treatment Facilities

**DOI:** 10.3390/ijerph18168884

**Published:** 2021-08-23

**Authors:** Fares Qeadan, Nana A. Mensah, Lily Y. Gu, Erin F. Madden, Kamilla L. Venner, Kevin English

**Affiliations:** 1 Department of Family and Preventive Medicine, University of Utah School of Medicine, Salt Lake City, UT 84108, USA; nana.mensah@utah.edu (N.A.M.); lily.gu@utah.edu (L.Y.G.); 2 Department of Family Medicine and Public Health Sciences, Wayne State University, Detroit, MI 48201, USA; efmadden@wayne.edu; 3 Department of Psychology, Center on Alcohol, Substance Use, and Addiction (CASAA), University of New Mexico, Albuquerque, NM 87131, USA; kamilla@unm.edu; 4 Albuquerque Area Southwest Tribal Epidemiology Center, Albuquerque, NM 87110, USA; kenglish@aaihb.org

**Keywords:** medication for addiction treatment, alcohol use disorder, TEDS-A, naltrexone

## Abstract

Background: Naltrexone, a medication for addiction treatment (MAT), is an FDA-approved medication recommended for the treatment of alcohol use disorder (AUD). Despite the high prevalence of AUD and efficacy of naltrexone, only a small percentage of individuals with AUD receive treatment. Objectives: To identify trends for the prescription of naltrexone in AUD admissions in substance use treatment centers across the U.S. Methods: Data from the 2000–2018 U.S. Treatment Episode Data Set: Admissions (TEDS-A) were used in temporal trend analysis of naltrexone prescription in admissions that only used alcohol. Data from the 2019 National Survey of Substance Abuse Treatment Services (N-SSATS) were also used to characterize medication use among AUD clients across different treatment service settings. Results: Treatment of AUD with naltrexone was 0.49% in 2000 and tripled from 0.53% in 2015 to 1.64% in 2018 in AUD admissions (*p* < 0.0001 for the Cochran–Armitage trend test). Women, middle-aged adults, and admissions for clients living in the Northeast U.S. were more likely to be prescribed naltrexone than their respective counterparts, as were admissions with prior treatment episodes and referrals through alcohol/drug use care providers, who paid for treatment primarily through private insurance, used alcohol daily in the month prior to admission, and waited 1–7 days to enter treatment. Naltrexone was more commonly prescribed by AUD admissions compared to acamprosate and disulfiram and was more frequently prescribed in residential and outpatient services as opposed to hospital inpatient services. Conclusions: Naltrexone remains underutilized for AUD, and factors that influence prescription of medication are multifaceted. This study may contribute to the creation of effective interventions aimed at reducing naltrexone disparities for AUD.

## 1. Introduction

### AUD Prevalence and Societal Costs

Alcohol use disorder (AUD) describes a problematic pattern of alcohol use characterized by an inability to stop or control alcohol intake despite adverse health outcomes or social or work consequences [1]. Current literature points to a high prevalence of AUD in the United States. In 2019, approximately 14.1 million adults aged 18 years and older suffered from AUD [2]. Indeed, the harmful use of alcohol constitutes a significant source of morbidity [3], including high blood pressure, heart disease, stroke, liver disease, and cancer [4]. In the same vein, alcohol-related mortality continues to pose a hefty public health threat. In the United States, alcohol-related deaths remain the third leading cause of preventable deaths, with an estimated 95,000 deaths annually [5]. Furthermore, alcohol misuse is associated with substantial economic and societal costs. For instance, in 2010, healthcare and lost productivity costs due to alcohol misuse totaled $249 billion [6].

Despite the high prevalence of AUD and subsequent societal costs, only a small proportion of people with AUDs receive any type of treatment. A 2015 study showed that among individuals with 12-month and lifetime AUD, a mere 7.7% and 19.8% sought treatment for AUD, respectively [7]. Historically, non-pharmacological treatment modalities that focus on behavioral and psychosocial approaches have been used to treat AUD [8]. A variety of these approaches have been shown to sustain abstinence and reduce alcohol consumption effectively [8,9]. The advent of pharmacological treatments for AUD provides alternative approaches to traditional modalities. However, pharmacological treatments for AUD continue to be underutilized [10,11] despite clear evidence of benefits [12]. Possible mechanisms that drive the limited uptake of pharmacotherapy for AUD may include a lack of awareness of medication efficacy and limited pool of physicians trained in addiction treatment [9,13]. Additionally, the intense focus on medications for addiction treatment (MAT) for Opioid Use Disorder (OUD) may have also created a silo effect that contributed to relative neglect for expanding access to pharmacotherapy for AUD [14]. Racial and ethnic disparities in AUD treatment and socioeconomic challenges further exacerbate problems with access to care [15].

In light of the efficacy and availability of Food and Drug Administration (FDA)-approved pharmacological treatments, many health-related U.S. agencies recommend using MAT for moderate to severe AUD [16,17]. MAT combines medications, counseling, and behavioral therapies to provide a holistic approach to treatment [18]. Naltrexone, a long-acting opioid receptor antagonist, is one of three MAT options approved by the FDA to treat AUD [17]. It is also the recommended first-line treatment for AUD [19]. Key benefits of naltrexone include reductions in alcohol cravings and decreased relapse to heavy alcohol consumption, although such effects are modest [20,21,22]. However, these benefits may be undermined by poor medication adherence [17,23]. In such cases, the use of extended-release naltrexone, an injectable long-acting form of naltrexone, may eliminate non-adherence concerns [24,25].

Although the underutilization of naltrexone for AUD has been documented in the literature [26,27], we are not aware of any studies that have specifically examined long-term trends in naltrexone use for AUD. Studies highlighting trends in naltrexone treatment often do so within the context of opioid use rather than alcohol use [28,29]. Moreover, studies that describe naltrexone treatment among AUD populations are limited by outdated data [30,31]. This study presents a historical overview of naltrexone prescription among AUD admissions to provide a comprehensive understanding of changes over time. Specifically, we provide data on the proportion of AUD admissions prescribed naltrexone in substance use treatment facilities from 2000 to 2019 using data from the U.S. Treatment Episode Data Set: Admissions (TEDS-A). Furthermore, we examine these trends by a variety of demographic and treatment variables.

## 2. Methods

### 2.1. Study Population

#### 2.1.1. Dataset 1: Treatment Episode Data Set: Admissions (TEDS-A)

Our analysis consisted of 2000–2018 data from the U.S. Treatment Episode Data Set: Admissions (TEDS-A), which provides yearly, cross-sectional national data on admissions to substance use disorder (SUD) treatment facilities. TEDS-A is maintained by the Center for Behavioral Health Statistics and Quality (CBHSQ) at the Substance Abuse and Mental Health Service Administration (SAMHSA). The primary sampling unit in TEDS-A is admission, not a client, and thus a client could be recorded in multiple admissions. Given that TEDS-A does not have identifiers, it was not possible to capture data longitudinally (i.e., we were not able to link admissions for the same client). TEDS-A captures admission data that are collected by the individual state administrative data systems to monitor their substance use disorder treatment systems. Facilities that are required to report to the state substance abuse agency (SSA) include those that (i) receive public funds and/or are (ii) licensed or certified by the SSA to provide substance use disorder treatment (or are administratively tracked for other reasons) [32]. TEDS-A data come from all 50 States, the District of Columbia, and Puerto Rico from all government-funded programs, and that includes about 60% of states reported data on all admissions to eligible facilities. All variables in TEDS-A are categorical. We restricted our study population to admissions reporting alcohol as their only substance of use and who therefore did not experience other SUDs other than AUD. Admissions with unknown, uncollected, invalid, or otherwise missing responses for the outcome variable were excluded from analysis.

#### 2.1.2. Dataset 2: National Survey of Substance Abuse Treatment Services (N-SSATS)

For an extra-analysis, in parallel to analyzing TEDS-A, we used 2019 data from the National Survey of Substance Abuse Treatment Services (N-SSATS), an annual census of SUD treatment facilities that collects data on services, structure, and utilization of SUD treatment in the U.S. N-SSATS is a nationally representative observational annual survey of U.S. public and private SUD treatment facilities for which data are collected by mail, phone, and web-based questionnaires. N-SSATS is maintained by CBHSQ at SAMHSA, and it collects three types of information from facilities: characteristics of individual facilities, client count information, and general information, such as certification. About 90% of facilities report complete survey to N-SSATS [33]. In addition to facilities approved by state substance abuse agencies, N-SSATS also includes programs operated by federal agencies, the Department of Veterans Affairs, the Department of Defense, and the Indian Health Service [34].

Since N-SSATS includes information at the facility level, and TEDS-A includes information at the admissions level, we first report our results from N-SSATS.

### 2.2. Measures

Using TEDS-A data, we determined whether a client was prescribed naltrexone (outcome variable) as part of his or her treatment plan based on a binary “Yes” or “No” response. Naltrexone (or extended-release naltrexone) was the only form of medication-assistant treatment that our strictly AUD client population would have been prescribed if they reported “Yes”. This indicator represents a proxy for actual naltrexone receipt since the dataset does not include indications on the extent to which naltrexone is actually implemented [35]. Demographic factors included biological sex (male or female), age at admission (divided into six categories from 12 to 65+ years), and region of treatment (Northeast, Midwest, South, West). U.S. territories (i.e., Puerto Rico) were excluded as a category for region due to limited sample size.

We adopted the conceptual framework for drug treatment process and outcomes from Simpson (2004) [36] to guide our selection of relevant predictors that might influence MAT reception. Under this framework, we included relevant patient attributes (i.e., frequency of alcohol use, prior history of substance use treatment episodes, treatment referral source) and treatment or program attributes (i.e., service setting, number of days waiting to enter treatment (i.e., the number of days from the first contact or request for service until the client was admitted and the first clinical service was provided), primary payment source for treatment) as stratifying variables in our modeling. Frequency of alcohol use was categorized as daily use, some use, or no use in the past month prior to admission. Prior history of treatment for substance use episodes in the past ranged from no prior episodes to five or more episodes. Source of treatment referral was categorized as alcohol or drug use care providers, other health care providers, self-referral or referral from family or friends, court or criminal justice referral (e.g., via driving under the influence (DUI)/driving while intoxicated (DWI) charges), or referrals from schools, employers, or the community (e.g., religious organizations, Alcoholics Anonymous, Al-Anon). Treatment or service setting at admission was divided into: hospital inpatient (detox, 24-h, hospital inpatient; rehab/residential, hospital non-detox), residential (rehab/residential, short term and long term; detox, 24-h, free-standing residential), and outpatient (ambulatory, including intensive outpatient, non-intensive outpatient, and detoxification). These specific groupings were used to match the same definitions of treatment service settings in N-SSATS data. Number of days waiting to enter treatment consisted of zero, 1–7, 8–14, 15–30, or 31+ days. Admissions’ primary source of payment (whether actual or expected) was divided into self-pay, private insurance (e.g., Blue Cross/BlueShield, workers’ compensation), Medicare, Medicaid, other government payments, no charge (e.g., free, charity, special research, teaching), and other forms of payment.

Using N-SSATS data, we examined variables related to AUD treatment in Section B of the 2019 survey, which involved the number of clients among facilities who received medications for AUD (i.e., acamprosate or Campral^®^, disulfiram or Antabuse^®^, and naltrexone) in different service settings (i.e., hospital inpatient, residential, and outpatient). Hospital inpatient services consisted of inpatient treatment and inpatient detoxification; residential services consisted of non-hospital treatment services, including long-term, short-term, and detoxification; and outpatient services consisted of regular outpatient treatment, intensive outpatient treatment, day treatment or partial hospitalization, detoxification, methadone/buprenorphine maintenance, or naltrexone treatment.

### 2.3. Statistical Analyses

Descriptive statistics (frequencies and proportions) were calculated for variables of interest in TEDS-A and N-SSATS. Using TEDS-A data, trend graphs illustrating the percentage of naltrexone prescription in AUD admissions from 2000 to 2018 were constructed, stratified by demographic variables and treatment-related characteristics. The Cochran–Armitage trend test was used to determine temporal changes in naltrexone reception from 2000 to 2018. All statistical analyses were conducted using SAS 9.4 (Cary, NC, USA) at a significance level of 0.05.

## 3. Results

From 2000 to 2018, there were a total of 7,406,716 AUD admissions in TEDS-A with a valid response to the naltrexone variable, with an average of 389,827 admissions per year. The smallest sample size per year occurred in 2018 (*n* = 286,485), while the largest sample size occurred in 2009 (*n* = 471,348). Across combined 2000 to 2018 data, AUD-only admissions in TEDS-A made up roughly 23% of all substance use treatment admissions. Among substance use treatment facilities in N-SSATS, over one-third in 2019 had up to 10% of their client population as strictly AUD clients (Table 1). Meanwhile, 13.41% of facilities saw 11 to 20% of their client population as strictly AUD, and nearly one in ten facilities saw more than 40% of their client population as strictly AUD.

**Figure 1:**
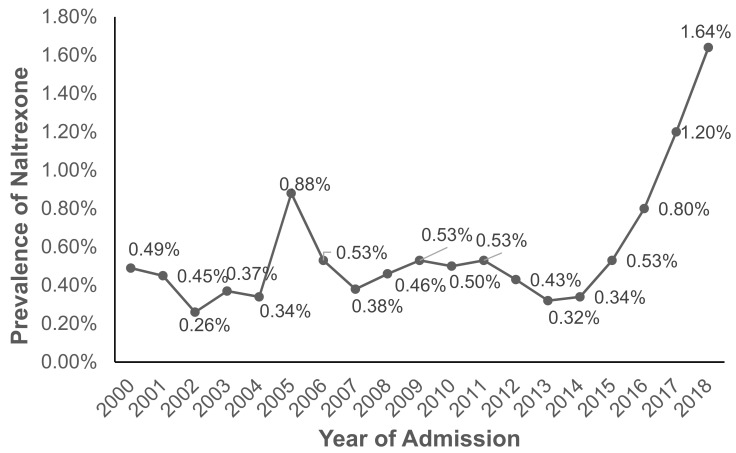
Prevalence of naltrexone treatment prescribed in alcohol use disorder (AUD) admissions from 2000–2018 using TEDS-A data. Temporal trends were significant by the Cochran–Armitage trend test (*Z* = −57.3, *p* < 0.0001).

**Figure 2:**
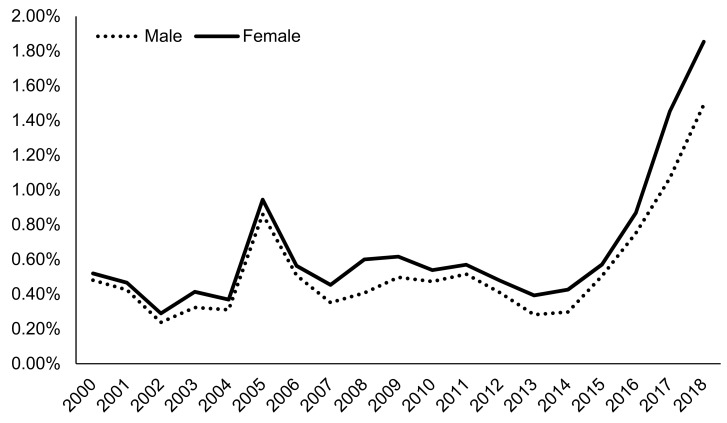
Prevalence of naltrexone treatment prescribed in alcohol use disorder (AUD) admissions from 2000–2018 by gender using TEDS-A data. Temporal trends were significant by the Cochran–Armitage trend test (*p* < 0.0001).

**Figure 3:**
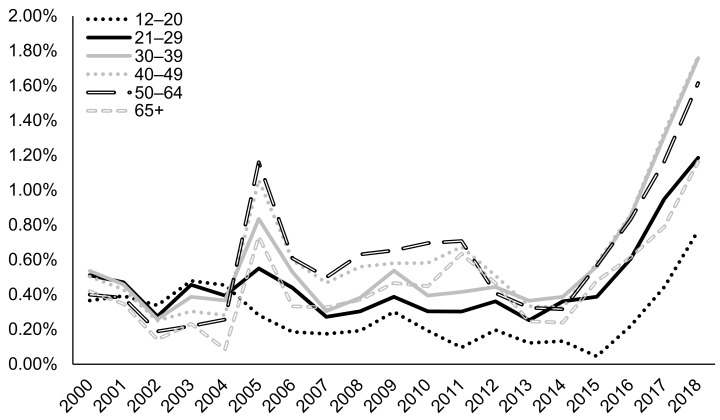
Prevalence of naltrexone treatment prescribed in alcohol use disorder (AUD) admissions from 2000–2018 by age at admission using TEDS-A data. Temporal trends were significant by the Cochran–Armitage trend test (*p* < 0.0001).

**Figure 4:**
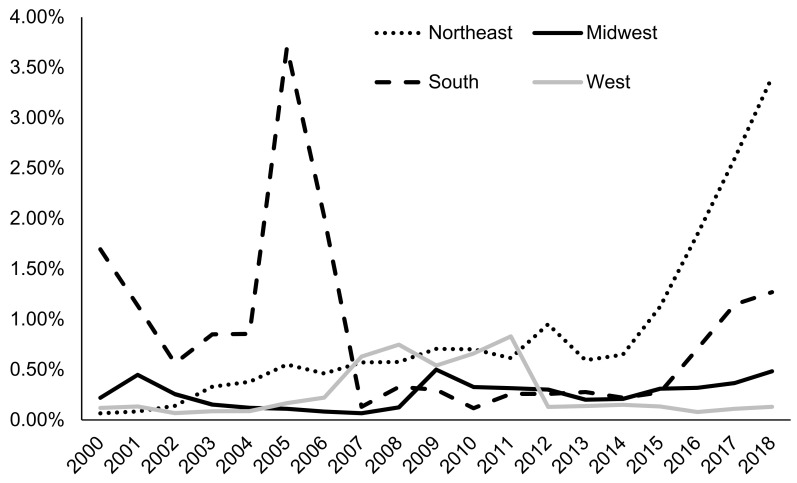
Prevalence of naltrexone treatment prescribed in alcohol use disorder (AUD) admissions from 2000–2018 by region of admission using TEDS-A data. Temporal trends were significant by the Cochran–Armitage trend test (*p* < 0.05).

According to 2019 N-SSATS, very few clients were prescribed any medication for AUD, and only a small fraction of facilities dispensed or prescribed AUD medication (Table 2). This trend is paralleled among TEDS-A regarding naltrexone as an AUD medication. In particular, treatment of AUD with naltrexone tripled from 0.53% in 2015 to 1.64% in 2018 in AUD admissions (*p* < 0.0001 for the Cochran–Armitage trend test; Figure 1). However, overall naltrexone treatment rates were low, with the most recent five-year average prevalence at 0.9%. Since 2000, females have been more likely to be prescribed naltrexone compared to males, with 1.85% of women being prescribed naltrexone in 2018 compared to 1.49% of men (Figure 2). Admissions for middle-aged clients, particularly those aged in their thirties to forties (~1.8% in 2018), followed by those aged in their fifties to mid-sixties (1.62% in 2018), were most likely to be prescribed naltrexone, while admissions for clients aged less than 20 were least likely to be prescribed naltrexone (0.77% in 2018) (Figure 3). Northeastern admissions were more likely to be prescribed naltrexone (3.40% in 2018) compared to Southern admissions (1.27% in 2018), Midwestern admissions (0.48% in 2018), and Western admissions (0.13% in 2018) (Figure 4). In particular, among Northeastern admissions, naltrexone prescription increased significantly since 2000, when the prevalence was just 0.07%. Meanwhile, this rate was relatively stable among Midwestern and Western admissions. Interestingly, Southern admissions saw an all-time high naltrexone prevalence of 3.70% in 2005.

**Figure 5:**
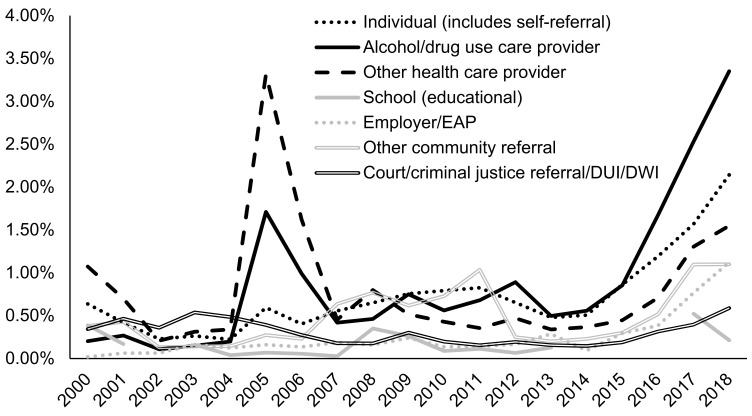
Prevalence of naltrexone treatment prescribed in alcohol use disorder (AUD) admissions from 2000–2018 by source of treatment referral using TEDS-A data. Temporal trends were significant by the Cochran–Armitage trend test (*p* < 0.05).

**Figure 6:**
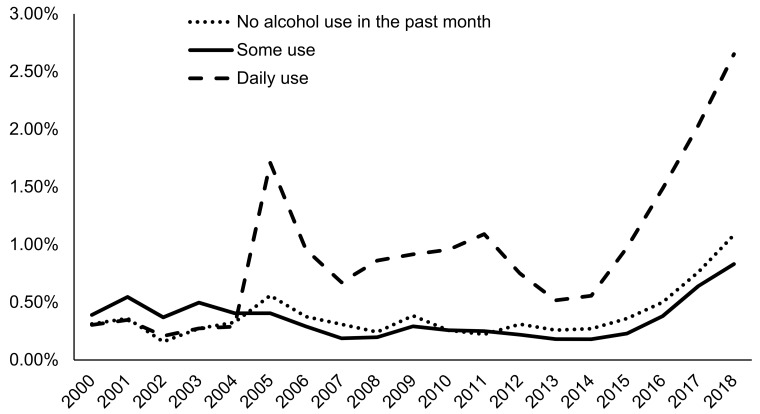
Prevalence of naltrexone treatment prescribed in alcohol use disorder (AUD) admissions from 2000–2018 by frequency of alcohol use at admission using TEDS-A data. Temporal trends were significant by the Cochran–Armitage trend test (*p* < 0.0001).

In general, AUD admissions with a greater number of prior treatment episodes were more likely to be prescribed naltrexone, with 4.05% of those having five or more prior episodes receiving naltrexone prescriptions in 2018 compared to only 0.81% of those with no prior episode history being prescribed naltrexone in 2018. Admissions for clients referred from alcohol/drug use care providers were most likely to be prescribed naltrexone (3.35% in 2018), followed by independently referred admissions (e.g., self-referral) (2.14% in 2018), and other health care provider referred admissions (1.55% in 2018) (Figure 5). Criminal justice and school-referred admissions had the lowest naltrexone rates at 0.59% and 0.21% in 2018, respectively. Admissions for clients who consumed alcohol daily at admission were more likely to be prescribed naltrexone (2.65% in 2018) compared to those who had no consumption in the past month (1.08% in 2018) or some (but less than daily) consumption (0.83% in 2018) (Figure 6).

**Figure 7:**
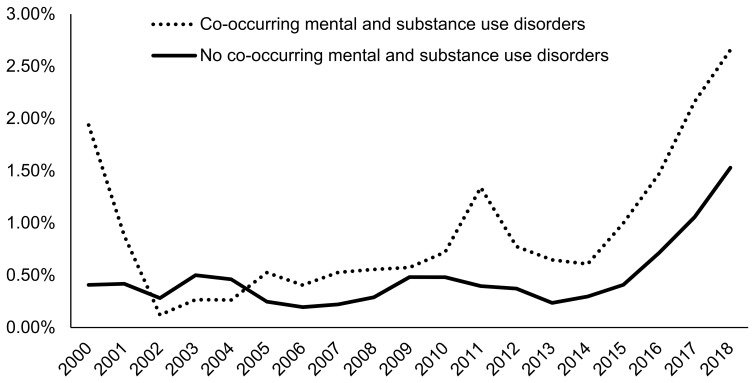
Prevalence of naltrexone treatment prescribed in alcohol use disorder (AUD) admissions from 2000–2018 by status of co-occurring mental disorders using TEDS-A data. Temporal trends were significant by the Cochran–Armitage trend test (*p* < 0.0001).

**Figure 8:**
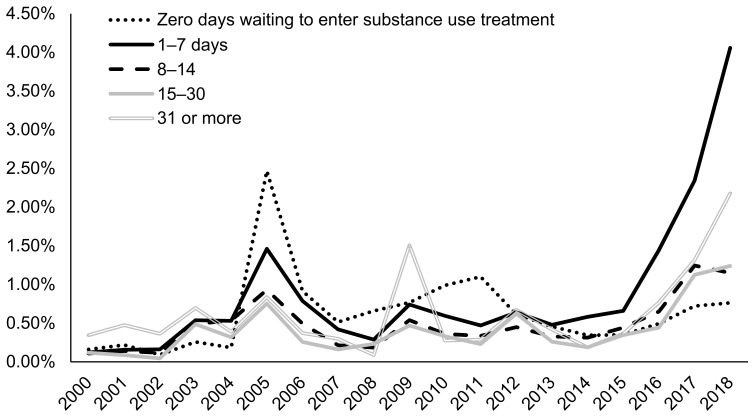
Prevalence of naltrexone treatment prescribed in alcohol use disorder (AUD) admissions from 2000–2018 by days waiting to enter treatment using TEDS-A data. Temporal trends were significant by the Cochran–Armitage trend test (*p* < 0.05).

**Figure 9:**
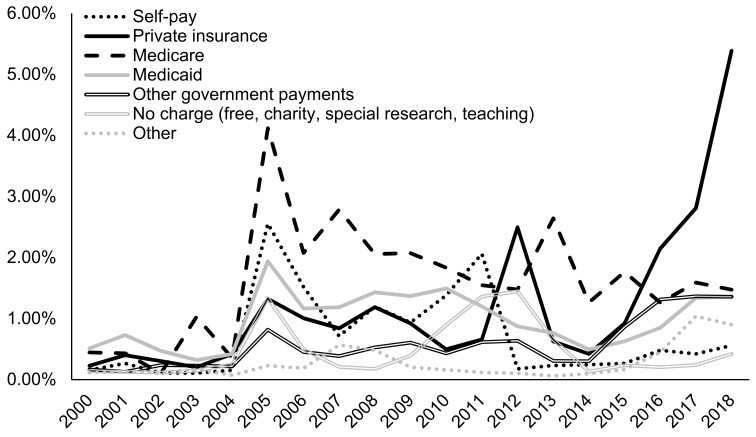
Prevalence of naltrexone treatment prescribed in alcohol use disorder (AUD) admissions from 2000–2018 by primary source of payment for treatment using TEDS-A data. Temporal trends were significant by the Cochran–Armitage trend test (*p* < 0.0001).

Since 2005, admissions with co-occurring mental disorders in conjunction with AUD (2.66% in 2018) were more likely to be prescribed naltrexone than AUD admissions with no mental disorders (1.53% in 2018) (Figure 7). Admissions for clients waiting one week or less to enter treatment were most likely to be prescribed naltrexone (4.06% in 2018), followed by admissions for clients waiting over one month to enter treatment (2.18% in 2018) (Figure 8). Admissions for clients waiting zero days to enter treatment had the lowest naltrexone rate at 0.76% in 2018. In recent years, admissions for clients who paid for treatment primarily through private insurance (5.39% in 2018) were most likely to be prescribed naltrexone compared to admission for clients paying through other methods, including out of pocket (0.56% in 2018), Medicare (1.47% in 2018), and Medicaid (1.36% in 2018) (Figure 9).

**Figure 10:**
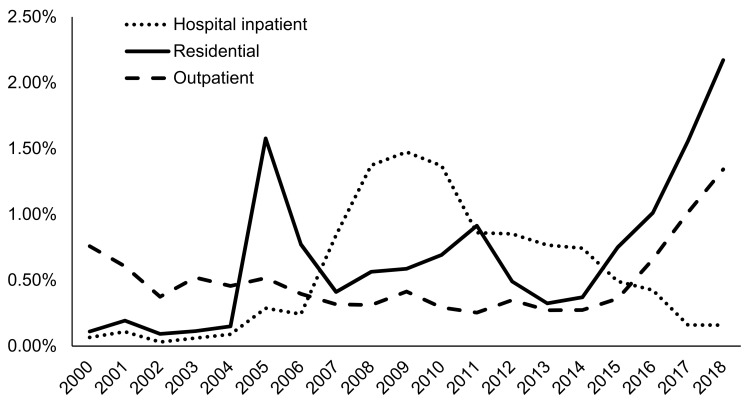
Prevalence of naltrexone treatment prescribed in alcohol use disorder (AUD) admissions from 2000–2018 by treatment service setting at admission using TEDS-A data. Temporal trends were significant by the Cochran–Armitage trend test (*p* < 0.0001). Hospital inpatient (detox, 24-h, hospital inpatient; rehab/residential, hospital non-detox); residential (rehab/residential, short term and long term; detox, 24-h, free-standing residential); outpatient (ambulatory, including intensive outpatient, non-intensive outpatient, and detoxification).

In 2018, nearly half of all AUD admissions in TEDS-A were for clients admitted to ambulatory, non-intensive outpatient services (43.20%), while one-quarter (24.36%) were for clients admitted to 24-h detoxification services at free-standing residential centers, and approximately one in ten were admitted to ambulatory, intensive outpatient facilities (11.11%) (Table 3). In recent years, AUD admissions for clients of residential service settings were more likely to be prescribed naltrexone (2.17% in 2018) compared to outpatient admissions (1.34% in 2018) (Figure 10). Hospital inpatient admissions had the lowest naltrexone prescription rate (0.16% in 2018). Overall, among N-SSATS facilities in 2019, AUD clients in outpatient services were most likely to be prescribed medication, followed by those in residential services, with very few AUD clients being prescribed medication in hospital inpatient services (Table 2). Naltrexone was the most commonly prescribed medication for AUD, with substantially more clients being prescribed naltrexone across all service settings compared to the other medications. In particular, 8.49% of N-SSATS facilities dispensed or prescribed naltrexone to two or more clients in outpatient settings.

## 4. Discussion

Our study draws on comprehensive data from TEDS-A and N-SSATS to highlight changes in naltrexone prescriptions among AUD admissions in U.S. treatment facilities from 2000 to 2018. Overall, we found a low but recently increasing prevalence of naltrexone among AUD admissions. More specifically, the percentage of AUD admissions receiving naltrexone prescriptions remained under 0.50% for much of the period between 2000 to 2014, followed by a sharp rise from 0.53% to 1.64% from 2015 to 2018. Additionally, we identified a host of disparities at specific time points, including sex, age, and geographic differences.

Like other recent studies [37,38,39], our analysis confirmed that the overall prevalence of naltrexone prescriptions in AUD admissions is low. However, we note that the follow-up rate in formal addiction treatment using naltrexone could reach about 15%, according to a recent study [40]. Considering that naltrexone for AUD constitutes evidence-based care and that it is the recommended first-line treatment for AUD, the relatively marginal increase in naltrexone prescriptions over the past two decades is worrisome. Although between 2015 and 2018, naltrexone use for AUD admissions tripled from 0.5% to 1.6%, still, this rate indicates a continuing underutilization of naltrexone for AUD. Interestingly, this period between 2015 and 2018 coincided with widespread acknowledgment of the national opioid crisis and increased government spending to combat opioid use disorders and overdose, including promotion of MAT for opioid use [41,42,43,44]. Other recent studies have suggested that the intense focus on mitigating the opioid crisis may have led to the neglect of other substance use problems [45]. Additionally, results from a recent cross-sectional study using the 2019 National Survey on Drug Use and Health (NSDUH) found that among an estimated 14.1 million adults with past-year AUD in 2019, only 1.6% used medications for treating AUD, and that included acamprosate, disulfiram, and naltrexone [46]. Our study demonstrated similar prevalence of 1.64% in 2018, however such prevalence was for being prescribed only naltrexone for treating AUD.

The potential reasons for sluggish naltrexone uptake has been the subject of several studies. These studies demonstrate that the reasons for the low uptake of naltrexone are varied, complex, and originate from several institutional and individual-level barriers [47,48,49,50,51]. Fundamental to the prompt adoption of any evidence-based practice are policies and procedures that promote accessibility and ease of use [52]. In the case of naltrexone, current health plan policies, such as complex pre-authorization stipulations, limitations on the number of medications allowed per prescription, and requirements to initiate lower level medications before naltrexone, may render naltrexone unattractive to both clinicians and patients [47]. Other institutional barriers may include treatment philosophy and practices, such as a focus on psychosocial treatments [53], inadequate availability of prescribing physicians, and staffing issues [47,49]. Similarly, individual-level barriers may stem from personal beliefs about the role of medications in alcohol addiction treatment and lack of knowledge about the efficacy of medications [51].

Although the factors mentioned above offer compelling reasons for the minimal increase in naltrexone use in AUD admissions, some observations in our dataset point to additional contributing factors. First, about a third of AUD admissions received acute detoxification services, which often constitutes cessation from alcohol use coupled with medically managed withdrawals. According to SAMHSA guidelines, naltrexone is not recommended prior to a complete detox from alcohol [17]. Therefore, it is possible that many admissions for clients entering detoxification services were not medically eligible for naltrexone. Additionally, more than 50% of AUD admissions from TEDS-A received outpatient treatment services in 2018. We found these rates consistent with 2019 data from N-SSATS. On the contrary, we also found that individuals receiving treatment in residential treatment settings were more likely to be treated with naltrexone in recent times. Although not impossible, naltrexone administration in outpatient treatment settings might be challenging for providers due to potential medication non-adherence commonly associated with oral naltrexone [54].

After stratification by key variables, we found significant disparities that are worth mentioning. One principal finding was that females consistently had a higher percentage for naltrexone prescription throughout the 18-year study period. Results from the literature regarding sex differences for alcohol use treatment are mixed [55]. There is evidence to support a shorter period between drinking initiation and seeking treatment among women [56,57] and an increased likelihood of treatment-seeking behavior for females than males [58]. Yet, other evidence points to treatment underutilization among women with AUD [59]. Our results are encouraging given that women present with harmful impacts of AUD far sooner than men [60], and yet, our findings underscore existing disparities among the sexes in receiving naltrexone as treatment for AUD.

Furthermore, our results uncovered prominent geographic differences in naltrexone prescription in AUD admissions. From 2015 onwards, admissions for clients who received treatment in the Northeast were more likely to be prescribed naltrexone than those who received treatment in other regions. One potential reason for these differences may be variation in state Medicaid expansion status at the time of data collection. As of 2018, all nine Northeastern states had elected to expand Medicaid compared to only 10 out of 13 states in the West [61]. Many state Medicaid programs cover a wide variety of SUD treatments, including naltrexone [62], so it follows that those states with expanded Medicaid programs are more likely to offer coverage for programs that use naltrexone.

Finally, our results showed significant differences in naltrexone trends by health insurance type. Specifically, we found that from 2015, naltrexone prescription in AUD admissions for clients with private health insurance more than tripled, while admissions for clients with other insurance types remained steady. Health plan coverage for AUD medications is essential in determining if patients receive naltrexone [63]. Thus, our findings support the need for insurers to cover medications such as naltrexone for AUD treatment.

### Limitations

Our results should be interpreted within the constraints of certain limitations. First, TEDS-A does not capture private facilities, and therefore, actual estimates may differ slightly. Certain predictors (i.e., number of days waiting for treatment and primary source of payment) had a large proportion of missing responses (making up to nearly 60% of all admissions), which could affect the accuracy of calculated stratified naltrexone prevalence. Despite this high proportion of missingness, our total non-missing sample sizes were substantially large for each year of data and allowed us to make approximations comparable to actual estimates. Further, the datasets contain only categorical information, which might have led to some loss of variability. We also note that it is not clear whether the number of prior treatment episodes was reported by the patient at the time of admission or was taken from what information the facility was aware of at admission from historical records. Nonetheless, we clarify that the number of prior treatment episodes was used in this study to identify severity of use, and hence, we do not expect the change from one definition to another will impact the significance of the results.

The AUD treatment variables in 2019 N-SSATS are a new development in the survey system, and there are current limitations to the data structure, such as being unable to distinguish between facilities that serve zero strictly AUD clients from those who serve at least one or more such clients. Similarly, we were unable to distinguish similar trends among facilities in regards to AUD medication uptake in different service settings. Future releases of N-SSATS may improve this section of the questionnaire.

## 5. Conclusions

Overall, we found a low prevalence of naltrexone prescriptions in AUD admissions. Additionally, we found different demographic and client characteristics and treatment-specific factors to be associated with uptake of naltrexone among admissions for AUD clients without other co-occurring substance use disorders. For instance, we found significant gender, age, and regional disparities in naltrexone prescription. Because the factors influencing the prescription of naltrexone are multifaceted, future research may investigate causal pathways contributing to the significant associations identified in these analyses. Such research may contribute to the creation of effective interventions aimed at reducing naltrexone disparities. Future studies should also use an intersectional approach to addressing disparities that considers individuals who fall into several risk categories that may further diminish the odds of receiving naltrexone prescription among alcohol and polysubstance use admissions.

The clinical implications of this study include the need for improving access to naltrexone for AUD and encouraging providers to prescribe naltrexone to treat AUD especially among those without co-occurring mental health diagnosis, those who do not use alcohol daily, those with smaller number of prior treatment episodes, younger patients, those who do not have a private insurance, and those who live in the Midwestern or the Western regions of the country. Other implications include the adaptation of approaches that increase health literacy about the benefits of naltrexone in treating AUD and others that eliminate systems-based barriers to prescribing naltrexone for AUD.

## Figures and Tables

**Table 1:** Distribution of substance use treatment facilities in 2019 by the percentage of clients treated for only alcohol abuse using N-SSATS data.

Percent of Clients Treated for Only Alcohol Abuse	*n* (%)
Not applicable	4046 (25.35%)
0–10	5456 (34.18%)
11–20	2140 (13.41%)
21–25	1098 (6.88%)
26–40	1542 (9.66%)
41+	1531 (9.59%)
Missing	148 (0.93%)

**Table 2:** Distribution of substance use disorder treatment facilities (hospital inpatient, residential, and outpatient) in 2019 by the total number of clients prescribed medication (acamprosate, disulfiram, and naltrexone) for alcohol use disorder using N-SSATS data.

Total Number of Clients Prescribed This Medication *	Hospital Inpatient	Residential	Outpatient
	*n* (%)	*n* (%)	*n* (%)
**Acamprosate (Campral^®^)**			
Not applicable	15,270 (95.67%)	12,907 (80.87%)	5968 (37.39%)
0 to 1	635 (3.98%)	2893 (18.13%)	9482 (59.41%)
2	15 (0.09%)	51 (0.32%)	134 (0.84%)
3 to 4	8 (0.05%)	36 (0.23%)	84 (0.53%)
5 or more	23 (0.15%)	44 (0.28%)	221 (1.38%)
Missing	10 (0.06%)	30 (0.19%)	72 (0.45%)
**Disulfiram (Antabuse^®^)**			
Not applicable	15,270 (95.67%)	12,907 (80.87%)	5968 (37.39%)
0 to 1	657 (4.12%)	2942 (18.43%)	9472 (59.34%)
2	8 (0.05%)	31 (0.19%)	144 (0.90%)
3 or more	19 (0.12%)	54 (0.34%)	307 (1.92%)
Missing	7 (0.04%)	27 (0.17%)	70 (0.44%)
**Naltrexone**			
Not applicable	15,270 (95.67%)	12,907 (80.87%)	5968 (37.39%)
0 to 1	547 (3.43%)	2580 (16.16%)	8563 (53.65%)
2 to 6	110 (0.69%)	339 (2.12%)	792 (4.97%)
7 or more	30 (0.19%)	107 (0.67%)	562 (3.52%)
Missing	4 (0.03%)	28 (0.18%)	76 (0.48%)

* Dispensed or prescribed at this facility for alcohol use disorder. % = column percentage out of total for each medication and service-setting combination. Outpatient (regular outpatient treatment, intensive outpatient treatment, day treatment or partial hospitalization, detoxification, methadone/buprenorphine maintenance, or naltrexone treatment); residential (non-hospital treatment services, including long-term, short-term, detoxification); hospital inpatient (inpatient treatment, inpatient detoxification).

**Table 3:** Distribution of admissions for alcohol use disorder (AUD) clients from 2018 TEDS-A data across different treatment service settings at admission, by receipt of naltrexone.

Treatment Service Setting at Admission	Naltrexone		
	Yes	No	Total
Detox, 24-h, hospital inpatient	18 (0.39%)	14,578 (5.17%)	14,596 (5.09%)
Detox, 24-h, free-standing residential	1387 (30.30%)	68,392 (24.26%)	69,779 (24.36%)
Rehab/residential, hospital (non-detox)	7 (0.15%)	1117 (0.40%)	1124 (0.39%)
Rehab/residential, short term (30 days or fewer)	758 (16.56%)	26,172 (9.28%)	26,930 (9.40%)
Rehab/residential, long term (more than 30 days)	258 (5.64%)	13,688 (4.86%)	13,946 (4.87%)
Ambulatory, intensive outpatient	287 (6.27%)	31,529 (11.18%)	31,816 (11.11%)
Ambulatory, non-intensive outpatient	1827 (39.92%)	121,924 (43.25%)	123,751 (43.20%)
Ambulatory, detoxification	35 (0.76%)	4508 (1.60%)	4543 (1.59%)
Total	100%	100%	100%

% are column percentages. Services were categorized into: hospital inpatient (detox, 24-h, hospital inpatient; rehab/residential, hospital non-detox); residential (rehab/residential, short term and long term; detox, 24-h, free-standing residential); outpatient (ambulatory, including intensive outpatient, non-intensive outpatient, and detoxification).

## Data Availability

Publicly available datasets were analyzed in this study. This data can be found here: https://wwwdasis.samhsa.gov/dasis2/teds.htm (accessed on 28 July 2021); https://wwwdasis.samhsa.gov/dasis2/nssats.htm (accessed on 28 July 2021).

## References

[1] National Institute on Alcohol Abuse and Alcoholism Alcohol Facts and Statistics. https://www.niaaa.nih.gov/publications/brochures-and-fact-sheets/alcohol-facts-and-statistics#:~:text=An%20estimated%2095%2C0005%20people,poor%20diet%20and%20physical%20inactivity.

[2] SAMHSA 2019 National Survey on Drug Use and Health (NSDUH). Table 5.4A—Alcohol Use Disorder in Past Year among Persons Aged 12 or Older, by Age Group and Demographic Characteristics: Numbers in Thousands, 2018 and 2019. https://www.samhsa.gov/data/sites/default/files/cbhsq-reports/NSDUHDetailedTabs2018R2/NSDUHDetTabsSect5pe2018.htm#tab5-4a.

[3] World Health Organization (2019). Global Status Report on Alcohol and Health 2018.

[4] Centers for Disease Control and Prevention Excessive Alcohol Use. https://www.cdc.gov/chronicdisease/resources/publications/factsheets/alcohol.htm.

[5] Centers for Disease Control and Prevention Alcohol Related Disease Impact (ARDI) Application. https://www.cdc.gov/ARDI.

[6] Sacks J.J., Gonzales K.R., Bouchery E.E., Tomedi L.E., Brewer R.D. (2015). 2010 national and state costs of excessive alcohol consumption. Am. J. Prev. Med..

[7] Grant B.F., Goldstein R.B., Saha T.D., Chou S.P., Jung J., Zhang H., Pickering R.P., Ruan W.J., Smith S.M., Huang B. (2015). Epidemiology of DSM-5 Alcohol Use Disorder: Results from the National Epidemiologic Survey on Alcohol and Related Conditions III. JAMA Psychiatry.

[8] Witkiewitz K., Litten R.Z., Leggio L. (2019). Advances in the science and treatment of alcohol use disorder. Sci. Adv..

[9] Ray L.A., Bujarski S., Grodin E., Hartwell E., Green R., Venegas A., Lim A.C., Gillis A., Miotto K. (2019). State-of-the-art behavioral and pharmacological treatments for alcohol use disorder. Am. J. Drug Alcohol Abus..

[10] Huskamp H.A., Reif S., Greenfield S.F., Normand S.-L.T., Busch A.B. (2020). Medication Utilization for Alcohol Use Disorder in a Commercially Insured Population. J. Gen. Intern. Med..

[11] Rubinsky A.D., Chen C., Batki S.L., Williams E.C., Harris A.H.S. (2015). Comparative utilization of pharmacotherapy for alcohol use disorder and other psychiatric disorders among U.S. Veterans Health Administration patients with dual diagnoses. J. Psychiatr. Res..

[12] Maisel N.C., Blodgett J.C., Wilbourne P.L., Humphreys K., Finney J.W. (2013). Meta-analysis of naltrexone and acamprosate for treating alcohol use disorders: When are these medications most helpful?. Addiction.

[13] Oliva E.M., Maisel N.C., Gordon A.J., Harris A.H. (2011). Barriers to use of pharmacotherapy for addiction disorders and how to overcome them. Curr. Psychiatry Rep..

[14] Robertson A.G., Easter M.M., Lin H., Frisman L.K., Swanson J.W., Swartz M.S. (2018). Medication-Assisted treatment for alcohol-dependent adults with serious mental illness and criminal justice involvement: Effects on treatment utilization and outcomes. Am. J. Psychiatry.

[15] Vaeth P.A.C., Wang-Schweig M., Caetano R. (2017). Drinking, Alcohol Use Disorder, and Treatment Access and Utilization among U.S. Racial/Ethnic Groups. Alcohol. Clin. Exp. Res..

[16] Reus V.I., Fochtmann L.J., Bukstein O., Eyler A.E., Hilty D.M., Horvitz-Lennon M., Mahoney J., Pasic J., Weaver M., Wills C.D. (2018). The American Psychiatric Association practice guideline for the pharmacological treatment of patients with alcohol use disorder. Am. J. Psychiatry.

[17] Substance Abuse and Mental Health Services Administration, National Institute on Alcohol Abuse and Alcoholism (2015). Medication for the Treatment of Alcohol Use Disorder: A Brief Guide.

[18] SAMHSA Medication-Assisted Treatment (MAT). https://www.samhsa.gov/medication-assisted-treatment.

[19] Leighty A.E., Ansara E.D. (2019). Treatment outcomes of long-acting injectable naltrexone versus oral naltrexone in alcohol use disorder in veterans. Ment. Health Clin..

[20] Jonas D.E., Amick H.R., Feltner C., Bobashev G., Thomas K., Wines R., Kim M.M., Shanahan E., Gass C.E., Rowe C.J. (2014). Pharmacotherapy for adults with alcohol use disorders in outpatient settings: A systematic review and meta-analysis. Jama.

[21] Kranzler H.R., Soyka M. (2018). Diagnosis and pharmacotherapy of alcohol use disorder: A review. Jama.

[22] Rösner S., Hackl-Herrwerth A., Leucht S., Vecchi S., Srisurapanont M., Soyka M. (2010). Opioid antagonists for alcohol dependence. Cochrane Database Syst. Rev..

[23] Dermody S.S., Wardell J.D., Stoner S.A., Hendershot C.S. (2018). Predictors of Daily Adherence to Naltrexone for Alcohol Use Disorder Treatment during a Mobile Health Intervention. Ann. Behav. Med..

[24] Swift R.M., Aston E.R. (2015). Pharmacotherapy for alcohol use disorder: Current and emerging therapies. Harv. Rev. Psychiatry.

[25] Murphy C.E., Wang R.C., Montoy J.C., Whittaker E., Raven M. (2021). Effect of extended-release naltrexone on alcohol consumption: A systematic review and meta-analysis. Addiction.

[26] Huhn A.S., Hobelmann J.G., Ramirez A., Strain E.C., Oyler G.A. (2019). Trends in first-time treatment admissions for older adults with alcohol use disorder: Availability of medical and specialty clinical services in hospital, residential, and outpatient facilities. Drug Alcohol Depend..

[27] Iheanacho T., Issa M., Marienfeld C., Rosenheck R. (2013). Use of naltrexone for alcohol use disorders in the Veterans’ Health Administration: A national study. Drug Alcohol Depend..

[28] Alderks C.E. (2017). Trends in the Use of Methadone, Buprenorphine, and Extended-Release Naltrexone at Substance Abuse Treatment Facilities: 2003–2015 (Update).

[29] Hadland S.E., Wharam J.F., Schuster M.A., Zhang F., Samet J.H., Larochelle M.R. (2017). Trends in Receipt of Buprenorphine and Naltrexone for Opioid Use Disorder among Adolescents and Young Adults, 2001–2014. JAMA Pediatr..

[30] Williams E.C., Gupta S., Rubinsky A.D., Glass J.E., Jones-Webb R., Bensley K.M., Harris A.H.S. (2017). Variation in receipt of pharmacotherapy for alcohol use disorders across racial/ethnic groups: A national study in the U.S. Veterans Health Administration. Drug Alcohol Depend..

[31] Spithoff S., Turner S., Gomes T., Martins D., Singh S. (2017). First-line medications for alcohol use disorders among public drug plan beneficiaries in Ontario. Can. Fam. Physician.

[32] Substance Abuse and Mental Health Services Administration, Center for Behavioral Health Statistics and Quality (2019). Treatment Episode Data Set (TEDS): 2017. Admissions to and Discharges from Publicly-Funded Substance Use Treatment.

[33] Substance Abuse and Mental Health Services Administration (2020). National Survey of Substance Abuse Treatment Services (NSSATS): 2019. Data on Substance Abuse Treatment Facilities.

[34] Mojtabai R. (2004). Which substance abuse treatment facilities offer dual diagnosis programs?. Am. J. Drug Alcohol Abus..

[35] Stahler G.J., Mennis J. (2020). The effect of medications for opioid use disorder (MOUD) on residential treatment completion and retention in the US. Drug Alcohol Depend..

[36] Simpson D.D. (2004). A conceptual framework for drug treatment process and outcomes. J. Subst. Abus. Treat..

[37] Knudsen H.K., Abraham A.J., Roman P.M. (2011). Adoption and implementation of medications in addiction treatment programs. J. Addict. Med..

[38] Schmidt L.A., Rieckmann T., Abraham A., Molfenter T., Capoccia V., Roman P., Gustafson D.H., McCarty D. (2012). Advancing recovery: Implementing evidence-based treatment for substance use disorders at the systems level. J. Stud. Alcohol Drugs.

[39] Volkow N.D., Frieden T.R., Hyde P.S., Cha S.S. (2014). Medication-assisted therapies—Tackling the opioid-overdose epidemic. N. Engl. J. Med..

[40] Anderson E.S., Chamberlin M., Zuluaga M., Ullal M., Hawk K., McCormack R., D’Onofrio G., Herring A.A. (2021). Implementation of Oral and Extended-Release Naltrexone for the Treatment of Emergency Department Patients with Moderate to Severe Alcohol Use Disorder: Feasibility and Initial Outcomes. Ann. Emerg. Med..

[41] Gladden R.M., Martinez P., Seth P. (2016). Fentanyl law enforcement submissions and increases in synthetic opioid–involved overdose deaths—27 states, 2013–2014. Morb. Mortal. Wkly. Rep..

[42] O’Donnell J.K., Gladden R.M., Seth P. (2017). Trends in deaths involving heroin and synthetic opioids excluding methadone, and law enforcement drug product reports, by census region—United States, 2006–2015. MMWR. Morb. Mortal. Wkly. Rep..

[43] O’Donnell J.K., Halpin J., Mattson C.L., Goldberger B.A., Gladden R.M. (2017). Deaths involving fentanyl, fentanyl analogs, and U-47700—10 states, July–December 2016. MMWR. Morb. Mortal. Wkly. Rep..

[44] Center for Disease Control and Prevention (2018). Opioid Overdose.Understanding the Epidemic. https://www.cdc.gov/opioids/basics/epidemic.html.

[45] Cicero T.J., Ellis M.S., Kasper Z.A. (2020). Polysubstance Use: A Broader Understanding of Substance Use during the Opioid Crisis. Am. J. Public Health.

[46] Han B., Jones C.M., Einstein E.B., Powell P.A., Compton W.M. (2021). Use of Medications for Alcohol Use Disorder in the US: Results from the 2019 National Survey on Drug Use and Health. JAMA Psychiatry.

[47] Abraham A.J., Roman P.M. (2010). Early adoption of injectable naltrexone for alcohol-use disorders: Findings in the private-treatment sector. J. Stud. Alcohol Drugs.

[48] Chokron Garneau H., Venegas A., Rawson R., Ray L.A., Glasner S. (2018). Barriers to initiation of extended release naltrexone among HIV-infected adults with alcohol use disorders. J. Subst. Abus. Treat..

[49] Finlay A.K., Ellerbe L.S., Wong J.J., Timko C., Rubinsky A.D., Gupta S., Bowe T.R., Burden J.L., Harris A.H. (2017). Barriers to and facilitators of pharmacotherapy for alcohol use disorder in VA residential treatment programs. J. Subst. Abus. Treat..

[50] Robertson A.G., Swartz M.S. (2018). Extended-release naltrexone and drug treatment courts: Policy and evidence for implementing an evidence-based treatment. J. Subst. Abus. Treat..

[51] Williams E.C., Achtmeyer C.E., Young J.P., Berger D., Curran G., Bradley K.A., Richards J., Siegel M.B., Ludman E.J., Lapham G.T. (2018). Barriers to and facilitators of alcohol use disorder pharmacotherapy in primary care: A qualitative study in five VA clinics. J. Gen. Intern. Med..

[52] DiClemente R.J., Salazar L.F., Crosby R.A. (2013). Health Behavior Theory for Public Health: Principles, Foundations, and Applications.

[53] Roman P.M., Abraham A.J., Knudsen H.K. (2011). Using medication-assisted treatment for substance use disorders: Evidence of barriers and facilitators of implementation. Addict. Behav..

[54] Stoner S.A., Arenella P.B., Hendershot C.S. (2015). Randomized controlled trial of a mobile phone intervention for improving adherence to naltrexone for alcohol use disorders. PLoS ONE.

[55] McHugh R.K., Votaw V.R., Sugarman D.E., Greenfield S.F. (2018). Sex and gender differences in substance use disorders. Clin. Psychol. Rev..

[56] Alvanzo A.A., Storr C.L., Mojtabai R., Green K.M., Pacek L.R., La Flair L.N., Cullen B.A., Crum R.M. (2014). Gender and race/ethnicity differences for initiation of alcohol-related service use among persons with alcohol dependence. Drug Alcohol Depend..

[57] Lewis B., Nixon S.J. (2014). Characterizing gender differences in treatment seekers. Alcohol. Clin. Exp. Res..

[58] Haughwout S.P., Harford T.C., Castle I.J., Grant B.F. (2016). Treatment Utilization Among Adolescent Substance Users: Findings from the 2002 to 2013 National Survey on Drug Use and Health. Alcohol. Clin. Exp. Res..

[59] McCaul M.E., Roach D., Hasin D.S., Weisner C., Chang G., Sinha R. (2019). Alcohol and Women: A Brief Overview. Alcohol. Clin. Exp. Res..

[60] Foster K.T., Hicks B.M., Iacono W.G., McGue M. (2014). Alcohol use disorder in women: Risks and consequences of an adolescent onset and persistent course. Psychol. Addict. Behav..

[61] Kaiser Family Foundation Status of State Medicaid Expansion Decisions: Interactive Map. https://www.kff.org/medicaid/issue-brief/status-of-state-medicaid-expansion-decisions-interactive-map/.

[62] Abraham A.J., Yarbrough C.R., Harris S.J., Adams G.B., Andrews C.M. (2021). Medicaid Expansion and Availability of Opioid Medications in the Specialty Substance Use Disorder Treatment System. Psychiatr. Serv..

[63] Alanis-Hirsch K., Croff R., Ford J.H., Johnson K., Chalk M., Schmidt L., McCarty D. (2016). Extended-Release Naltrexone: A Qualitative Analysis of Barriers to Routine Use. J. Subst. Abus. Treat..

